# Supplementary material for: RNAmetasome network for macromolecule biogenesis in human cells
Source: Commun Biol. 2021 Dec 15;4:1399. doi: 10.1038/s42003-021-02928-y (PMC8674265; doi:10.1038/s42003-021-02928-y)
Supplement: Supplementary file 2 — Supplementary Information [file 42003_2021_2928_MOESM2_ESM.pdf]

RNAmetasome network for macromolecule biogenesis in human cells

Shiro Iuchi[1,✉] and Joao A. Paulo[1]

[1]Department of Cell Biology, Harvard Medical School,
240 Longwood Avenue,
Boston MA 20115.

✉ E-mail: shiro_iuchi@hms.harvard.edu

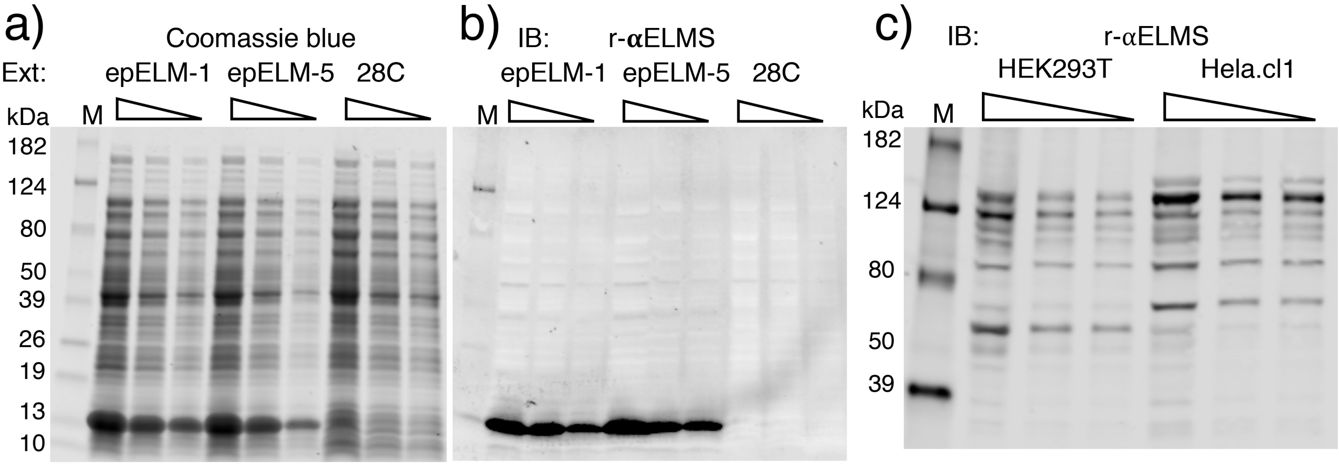

Supplementary Figure 1. Epitope for the rabbit anti-ELMSAN1 antibody. **a)** epELM-1and ep-ELM-5, which have a 6x His tagged epitope (^{550}AA^{600} of ELMSAN1, Q6PJG2), were produced in the presence of 0.5 mM IPTG in E. coli, and these epitopes were separated by SDS-PAGE. 28c is the control performed with the empty vector, pET28C (+). Thirty, 15, and 7.5 µg protein of the extracts was applied to a well from the left to the right. epELM-1 and epELM-5 were estimated to be 15% and 17% of the total proteins. **b)** Western blotting of the epitopes. The epitopes transferred to PVDF membrane were probed with the ani-ELMSAN1 antibody. A piece of PVDF with epELM-5 was excised from a replica of the IB and used to examine specificity of the anti-ELMSAN1 antibody (See Figure 1e). **c)** Western blot of the HEK293T and the Hela.cl1nuclear extract for ELMSAN1. Thirty, 15, and su7.5 µg protein of the nuclear extracts was applied to a well from the left to the right.

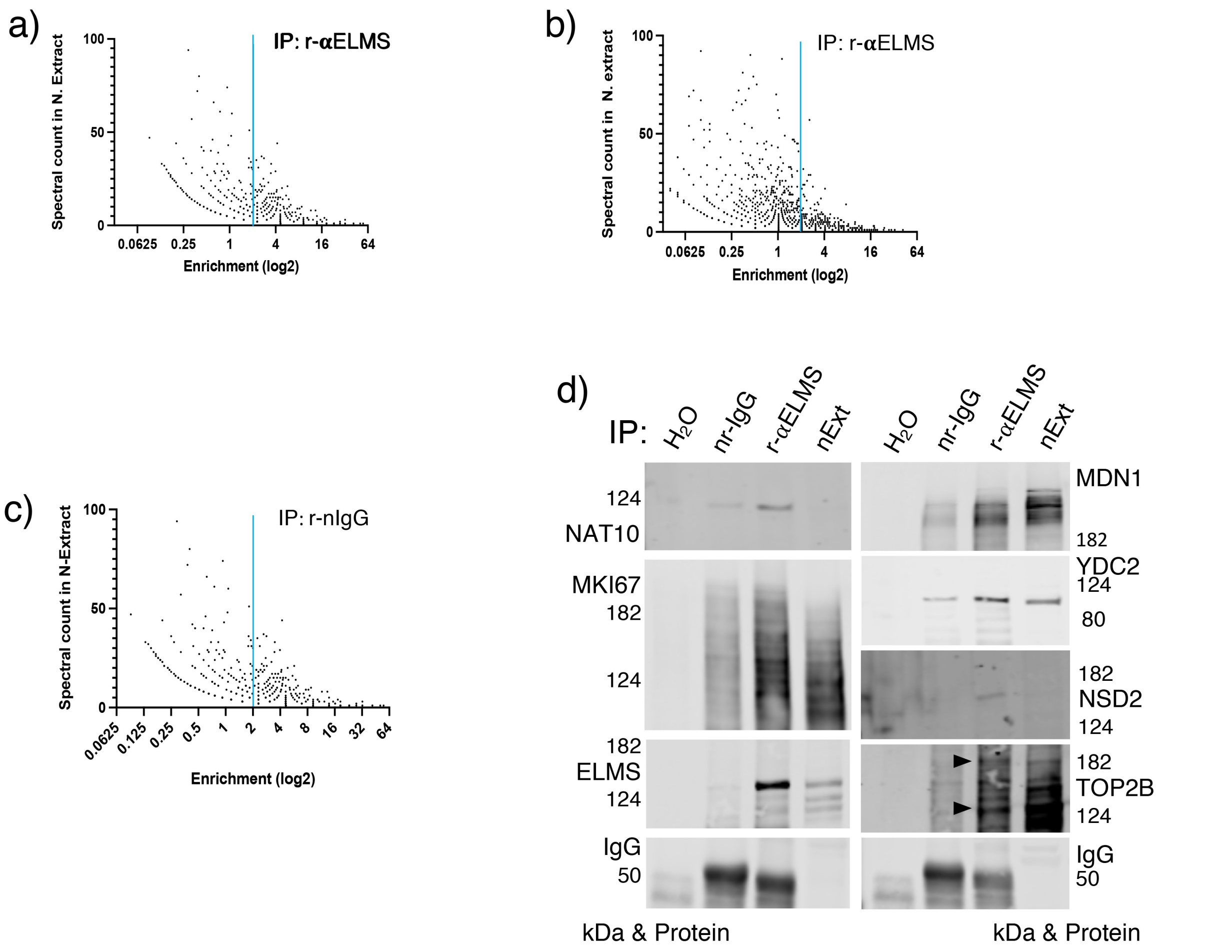

Supplementary Figure 2. IP-MS of the HEK293T nuclear extract. a), b) IP-MS of proteins precipitated with the anti-ELMSAN1 antibody. Y axis, protein concentration in the nuclear HEK293T extract; X axis, enrichment of proteins by the IP. **c)** IP-MS of the control performed with nr-IgG. **d)** IP-IB of selected proteins precipitated with the anti-ELMSAN1antibody. Two controls (water and nr-IgG) were conducted along with the IP. Although the normal IgG precipitated proteins, any of the precipitates was less than that with the anti-ELMSAN1 antibody.

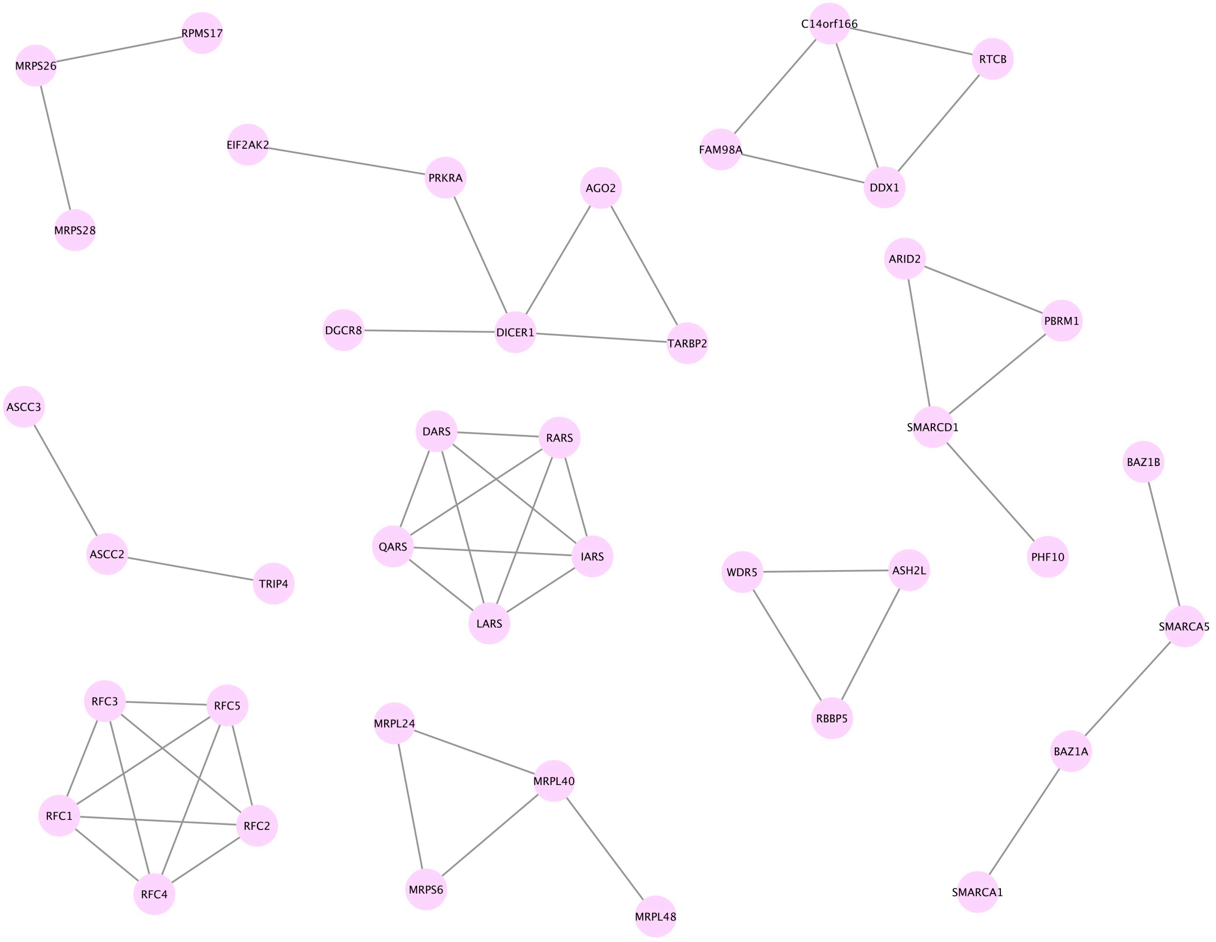

Supplementary Figure 3. Subgroups (modules) of proteins co-immunoprecipitated with the anti-ELMSAN1 antibody. These modules consist of proteins with multiple edges, but do not have an edge connecting them to the main RNAmetasome network.

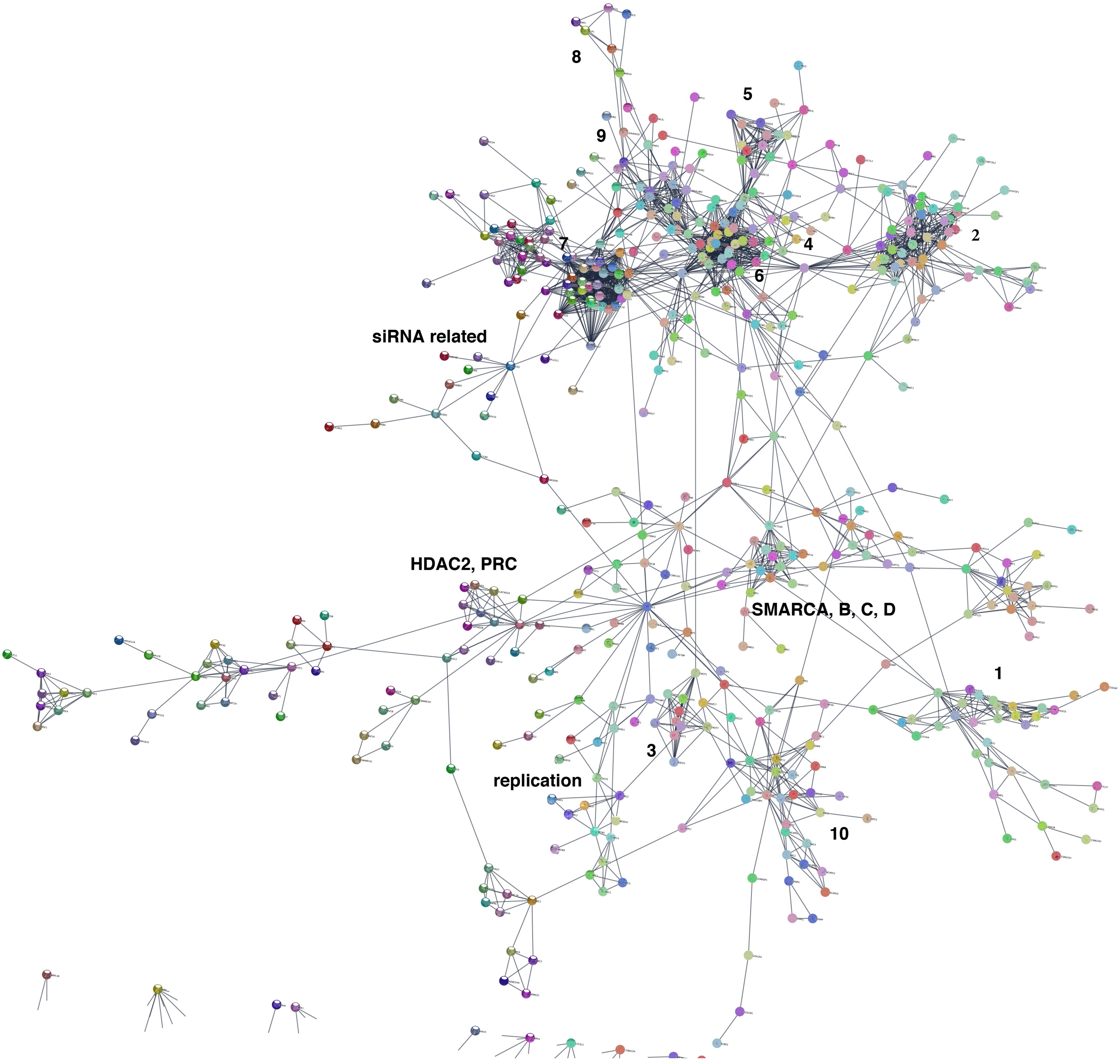

Supplementary Figure 4. Analysis of the IP-MS data (Exp #1 and #2) by SAINT and the network drawn by Cytoscape-STRING. These modules are labelled as numbered in Figure 2. Additional modules are spelled out.

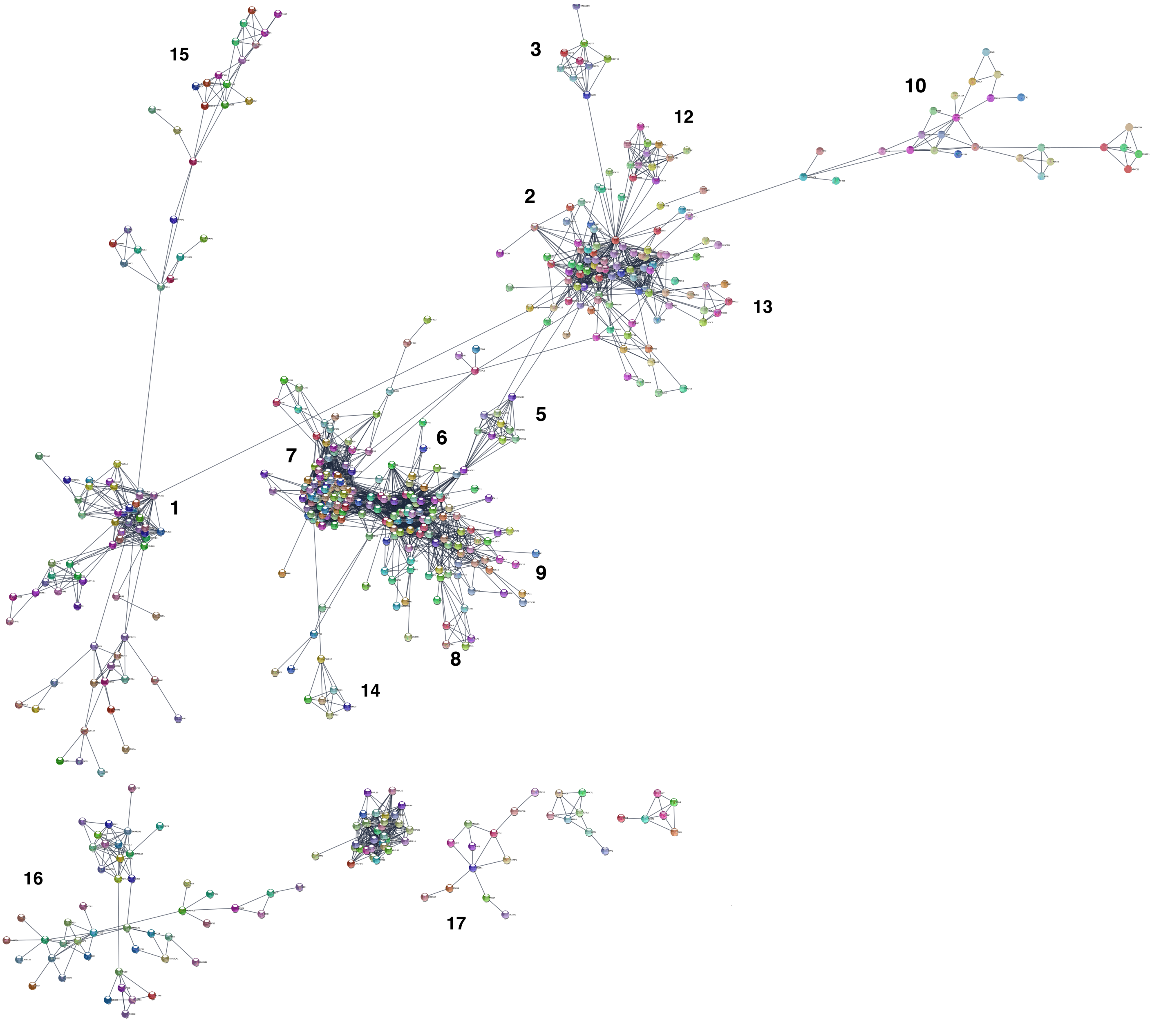

Supplementary Figure 5. RNAmetasome network obtained with another duplicate IP-MS (Exp #3 and #4). The data were analysed by SAINT and its network was drawn by Cytoscape-STRING. Modules are labelled as numbered in Figure 2. Additional labels (#13-17) indicate modules for degradation of mRNA (#12), export of mRNA, polyadenylated and spliced RNA (#13), and 26S proteasome (#14), DNA replication (#15) chromatin remodelling complexes (#16), and siRNA producing protein group (#17), respectively.

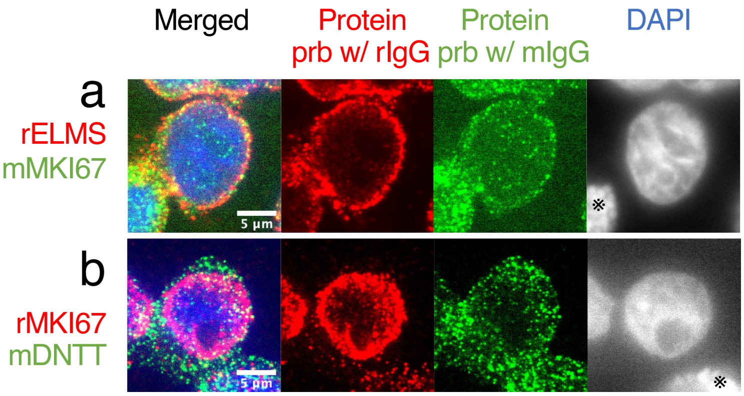

Supplementary Figure 6. Co-localisation of ELMSAN1 and DNTTIP1 with MKI 67.
※ indicates mitotic cell nucleus.

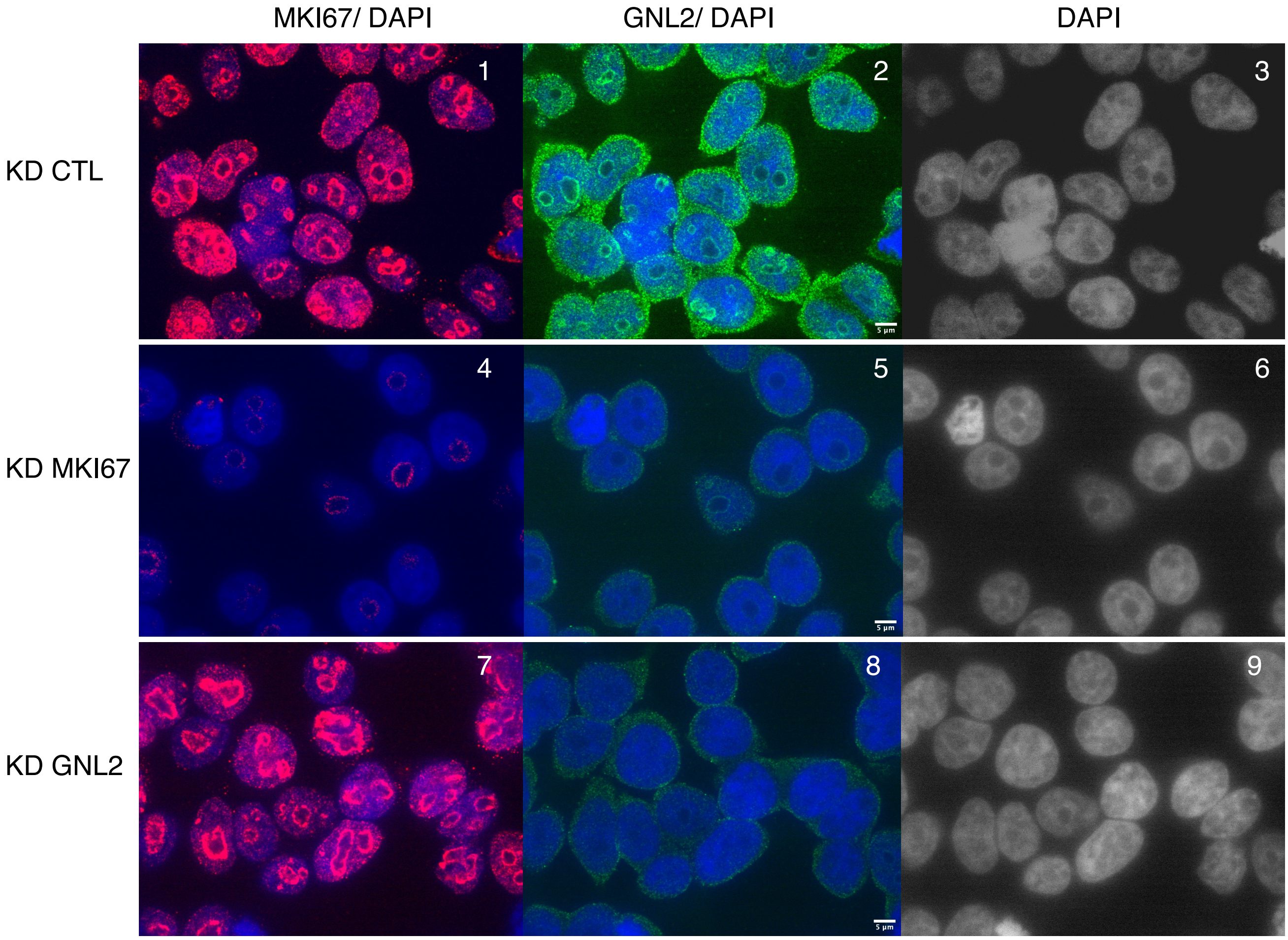

Supplementary Figure 7. Effect of the *MKI67* and the *GNL2* gene knockdown. Red, MKI67; green, GNL2; blue, DAPI. DsiRNA was added to the cultures at 10 nM.

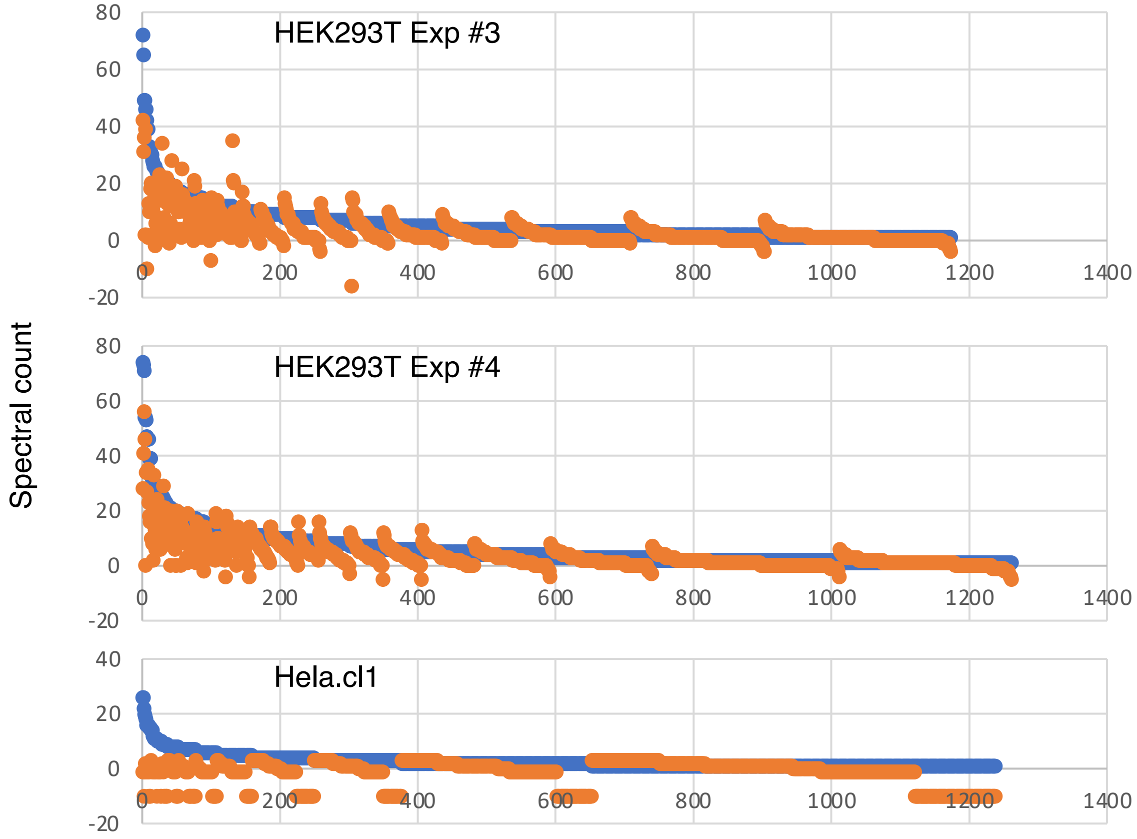

Supplementary Figure 8. Specific and "non-specific" binding of RNAmetasome network proteins. Spectral count on Y axis is difference from the control value with water. Blue, proteins precipitated with the anti-ELMSAN1 antibody; Orange, proteins precipitated with the nr-IgG. X axis shows sorting in which the anti-ELMSAN1 antibody precipitated proteins are plotted in order of the most to the left and the least to the right. The nr-IgG precipitated proteins are paired to that with the anti-ELMSAN1 antibody. HEK293T duplicate (Exp #3, #4) and Hela.cl1 experimental data are separately presented.

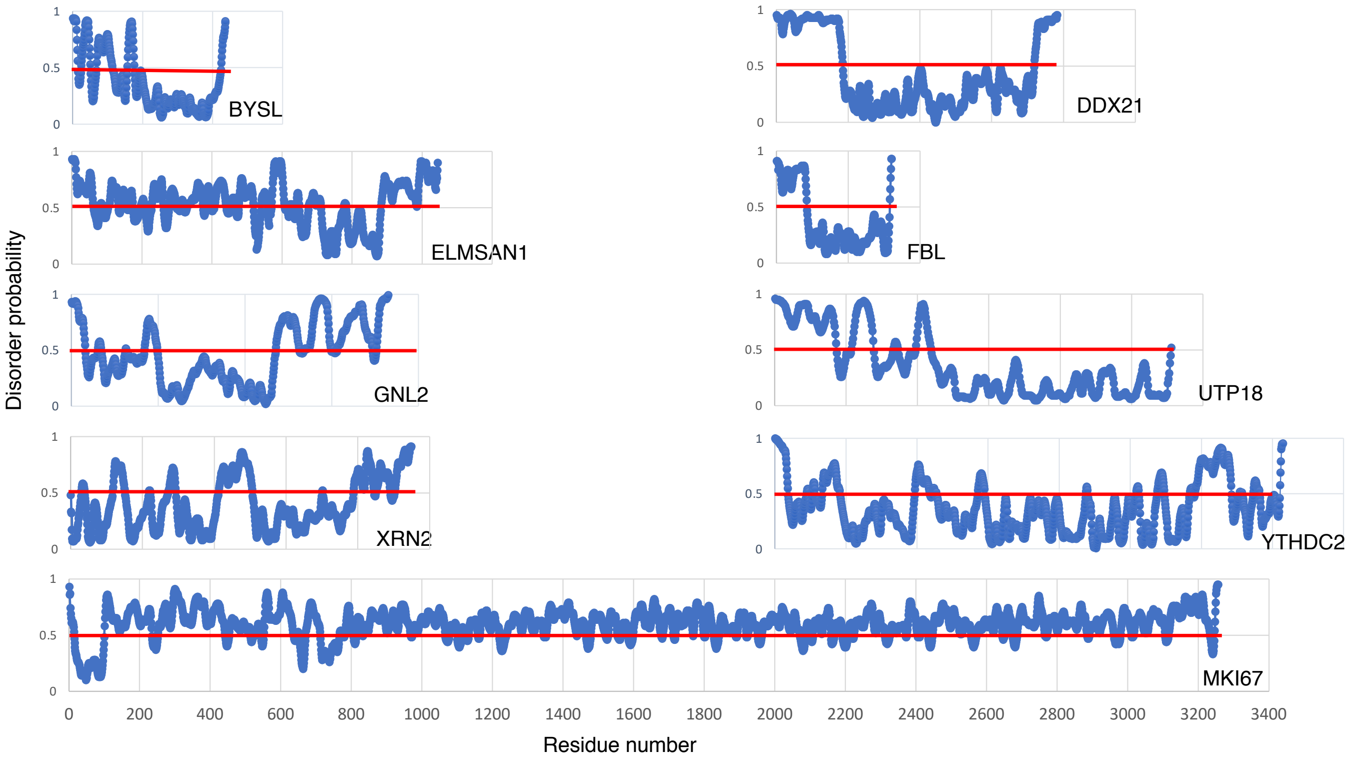

Supplementary Figure 9. Prediction of IDRs for 9 RNAmetasome network proteins. Red line shows threshold of the probability.

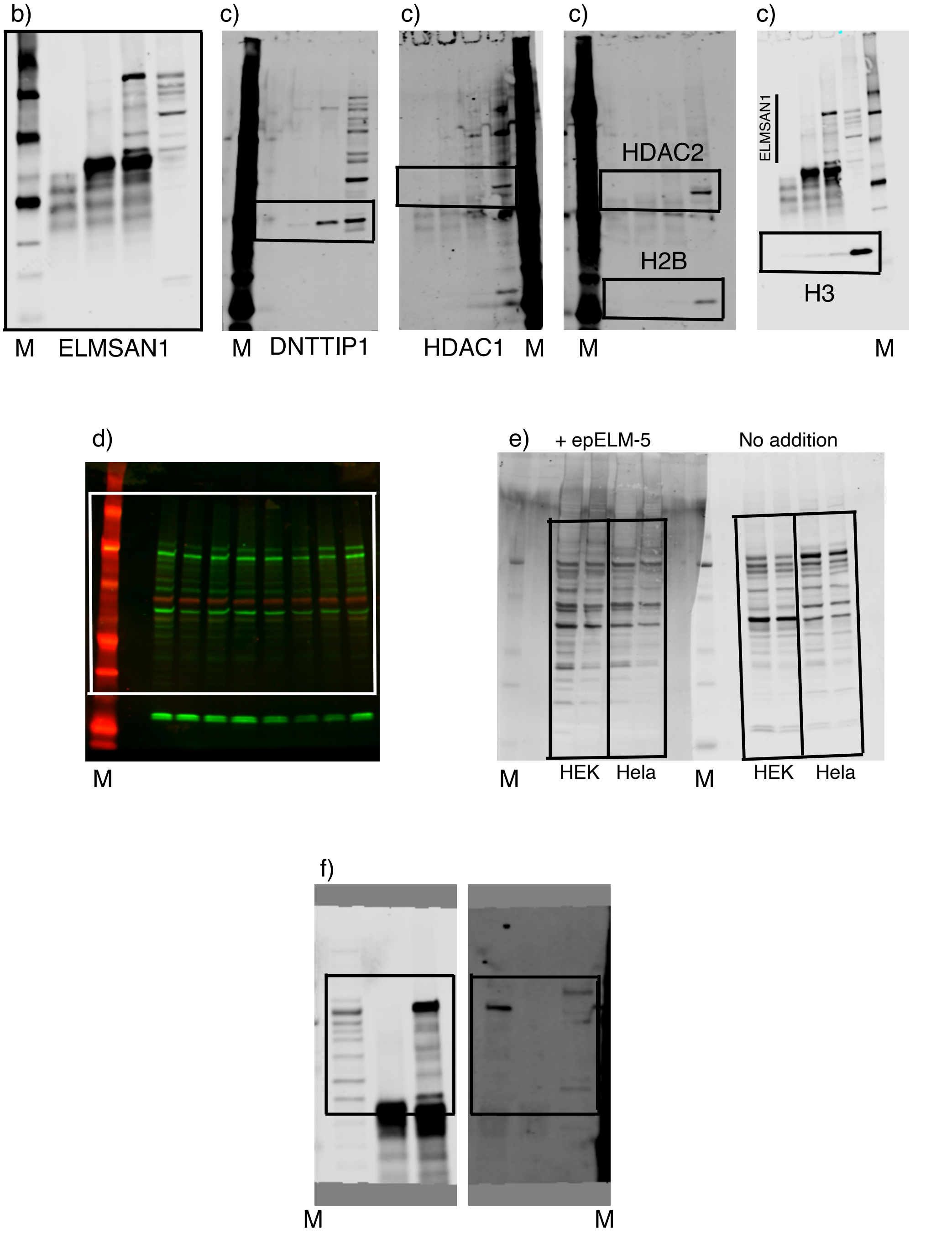

Supplementary Figure 10. Uncropped IB images of Figure 1. A cropped square of the Western blot has been presented in Figure 1. **b)-c)** Co-immunoprecipitation with the r-anti ELMSAN1 antibody. HDAC2 and H2B were simultaneously probed with the m-anti HDAC2 and the m-anti H2B antibodies, and H3 was probed with the r-anti H3 antibody using a membrane that was probed previously for ELMSAN1. **d)** Knockdown of the *ELMSAN1* gene. **e)** Excess epitope (ep-ELM-5) inhibited binding of the r-anti ELMSAN1 antibody to ELMSAN1 proteins. **f)** ELMSAN1 was probed with the r-anti ELMSAN1 (left) and the m-anti ELMSAN1 (right) antibodies. M, marker.

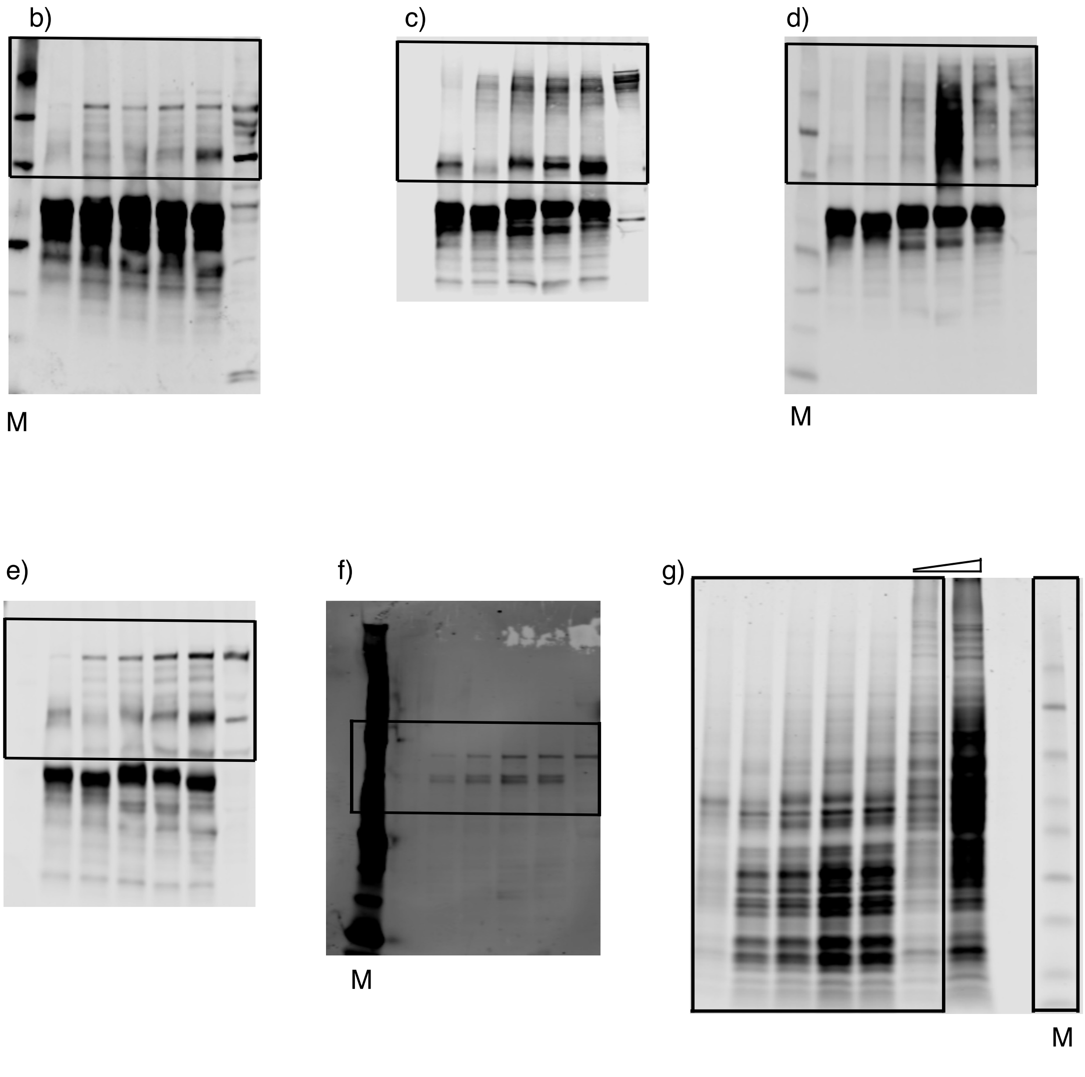

Supplementary Figure 11. Uncropped IB images of Figure 3, reciprocal immunoprecipitation. A cropped square of the Western blot has been presented in Figure 3. Each lane shows proteins precipitated with r-nIgG; r-anti ELMSAN1, r-anti-MDN1, r-anti MKI67, or r-anti YTHDC2 antibodies; or nuclear extract proteins (from the left to the right). These proteins are probed with **b)** r-anti ELMSAN1, **c)** r-anti-MDN1, **d)** r-anti MKI67, **e)** r-anti YTHDC2, and **f)** m-anti GNL2 antibodies. **g)** Coomassie blue staining of the immunoprecipitates. Right lane under the triangle shows result with greater amount of the nuclear extract. M, marker.

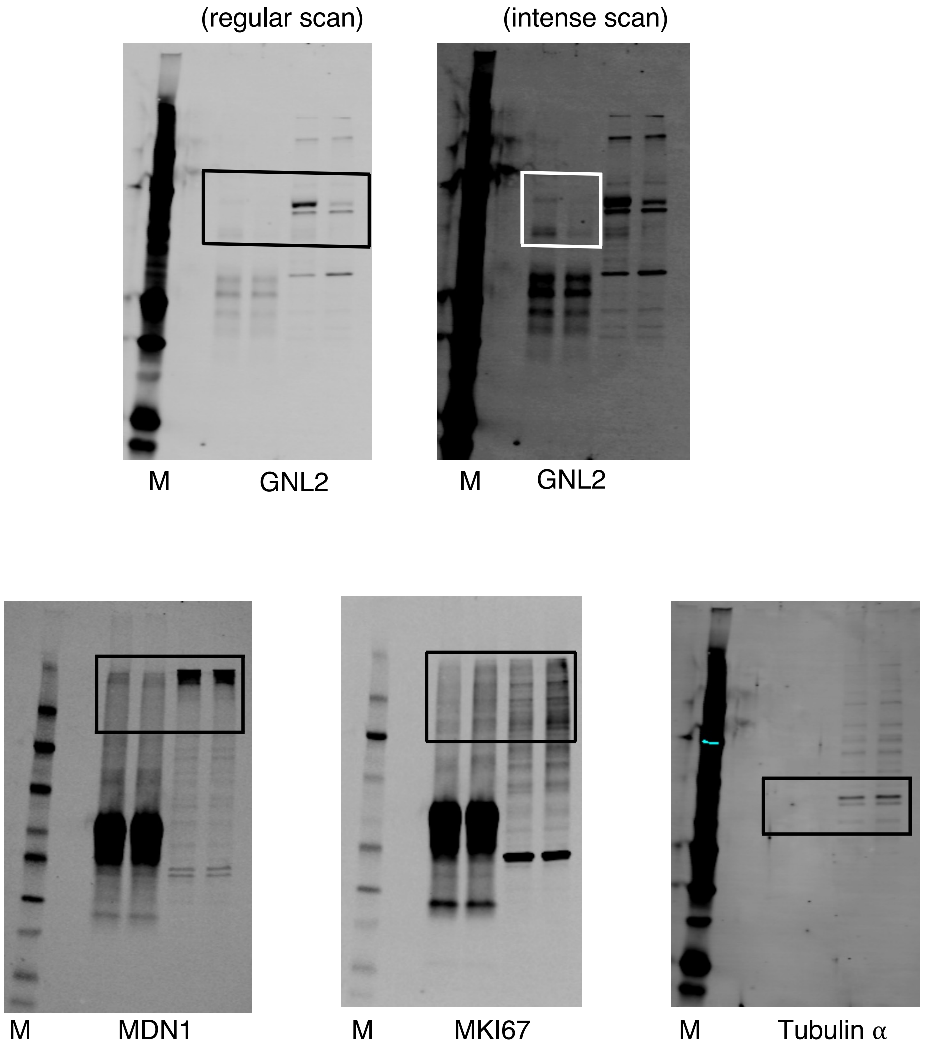

Supplementary Figure 12. Uncropped IB images of Figure 5, coimmunoprecipitation of GNL2 and MKI67 with the r-anti MDN1 antibody using GNL2 depleted nuclear extracts. A cropped square of the Western blot has been presented in Figure 5d. Left 2 lanes and right 2 lanes show results of IP and nuclear extracts, respectively. Effects of GNL2 depletion are shown in the right lane within the pair lanes. M, marker.

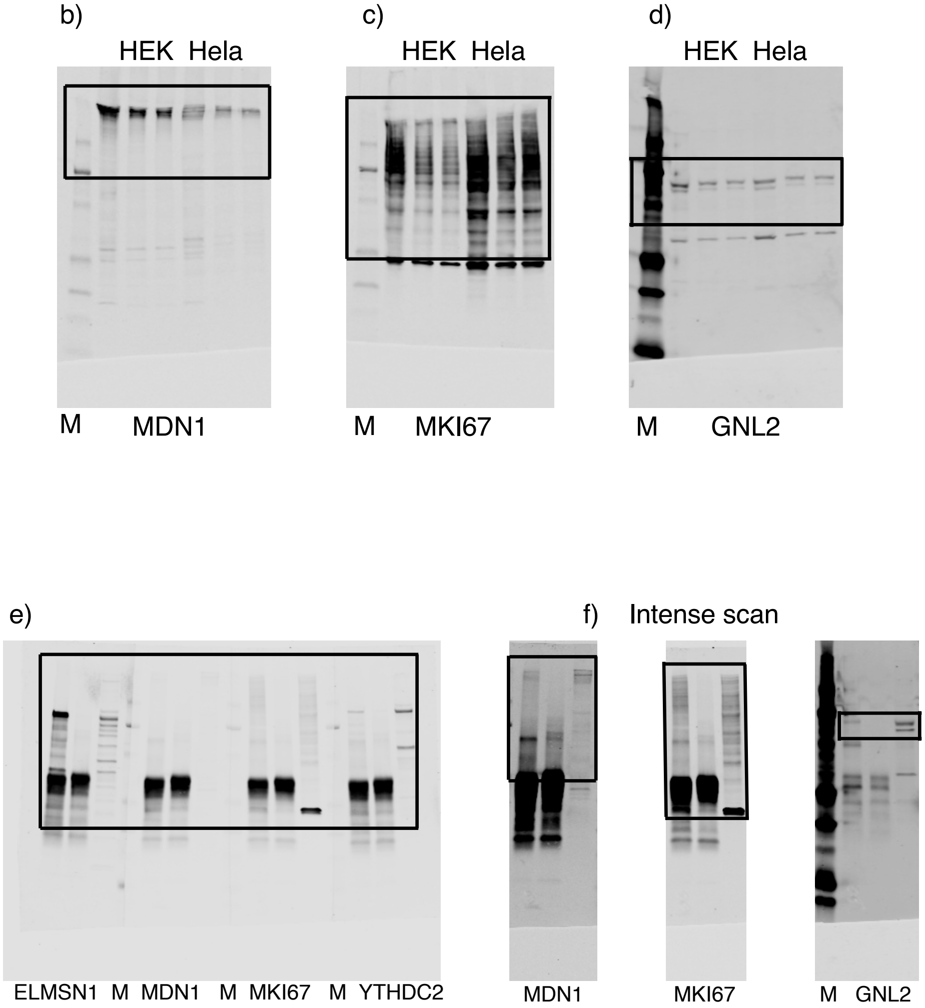

Supplementary Figure 13. Uncropped IB images of Figure 6, RNAmetasome proteins of Hela.cl1. A cropped square of the Western blot has been presented in Figure 6. **b)-d)** level of MDN1, MKI67, and GNL2 in Hela.cl1 nuclear extracts is shown compared with that in HEK293T extracts. **e)-f)** Co-immunoprecipitation of MDN1, MKI67, YTHDC2, and GNL2 with the r-anti ELMSAN1 antibody. M, marker.

Supplementary Table 1. antibodies used in this work.

Protein	Antibody	Host and Type	Concentration	(μg/ ml)	Cat#	Vendor
-	-	-	IF	WB	-	-
DDX20	Gemin3 (12H12)	m-Monoclonal	8	-	sc-57007	SANT CRUZ
DICER1	Dicer antibody(GT1176)	m-Monoclonal	5	-	MA527827	Thermo Fisher
NSD2	NSD2 (G-12)	m-Monoclonal	-	1	sc-365627	SANT CRUZ
DNTTIP1	TdTIF1 (G-2)	m-Monoclonal	-	1	sc-166296	SANT CRUZ
EIF3M	EIF3M antibody	m-Monoclonal	6.8	-	66025-1-Ig	proteintech
ELMSAN1	C14orf43 antibody	r-Polyclonal	1	0.25	A303-157A	BETHYL
ELMSAN1	ELMSAN1 (B-10)	m-Monoclonal	8	-	sc-514710	SANT CRUZ
GNL2	GNL2 (B-8)	m-Monoclonal	-	1	sc-514050	SANT CRUZ
NAT10	NAT-10 (B-4)	m-Monoclonal	-	1	sc-271770	SANT CRUZ
H2B	Purified anti-H2B	m-Polyclonal	-	0.5	688702	Biolegend
H3	Histone H3 antibody	r-Polyclonal	-	0.13	PA-16183	Thermo Fisher
HDAC1	HDAC1 (10E2)	m-Monoclonal	-	1	sc-81598	SANT CRUZ
HDAC2	HDAC2 (C-8)	m-Monoclonal	-	1	sc-9959	SANT CRUZ
MDN1	MDN1 antibody	r-Polyclonal	0.7	0.25	PA556225	Thermo Fisher
MKI67	Human Ki-67/MKI67 antibody	r-Monoclonal	1	0.25	MAB7617-SP	R&D
MKI67	Ki-67 (Ki67)	m-Monoclonal	8	-	sc-23900	SANT CRUZ
NIFK	NIFK (18E148)	m-Monoclonal	8	-	sc-52904	SANT CRUZ
YTHDC2	YTHDC2	r-polyclonal		1	27779-1-AP	proteintech
**α**-Tubulin	**α**Tubulin (B-7)	m-Monoclonal	-	1	sc-5286	SANT CRUZ
-	Normal Rabbit IgG	r-Polyclonal	1	0.25	12-370	Millipore
-	Normal mouse IgG	m-Polyclonal	8	1	sc-2025	SANT CRUZ
mouse IgGs, IgM, IgA	IRDye 680RD Goat anti-Mouse IgG	goat-Polyclonal	-	0.025	926-689070	LI-COR
rabbit IgG	IRDye 800 Affinity Purified Goat anti-Rabbit IgG	goat-Polyclonal	-	0.05	611-132-122	ROCKLAND
mouse IgG (H+L)	Goat anti-Mouse Highly Cross-Absorbed, Alexa Fluor 488	goat-Polyclonal	1	-	A11029	Thermo Fisher
rabbit IgG (H+L)	Goat anti-Rabbit Highly Cross-Absorbed, Alexa Fluor 555	goat-Polyclonal	1	-	A21429	Thermo Fisher

Supplementary Table 2. Nucleotide sequences used in this study.

Name	Object	DNA or DsiRNA (DsiR)	Sequence
Olit351	Epitope cloning	forward	5'-GGTCGGATCCTTGACGGGAAGGGTCC-3'
Olit352	Epitope cloning	reverse	5'-ACCGCAAGCTTATGGTTTCCGCACGG-3'
hs.Ri.ELMSNAN1.13.1	ELMSAN1 Knockdown	DsiR sense DsiR antisense	5'-GAGGUUGAAGUGGAUAUUAAGACTT-3' (3'-UU-5')
hs.Ri.ELMSNAN1.13.2	ELMSAN1 Knockdown	DsiR sense DsiR antisense	5'-AGCUCCUUGAGAAGUGAUAAACACC-3' (3'-UC-5')
hs.Ri.ELMSNAN1.13.3	ELMSAN1 Knockdown	DsiR sense DsiR antisense	5'-GGGAUGUGGAUACGAGCGAUGAGAA-3' (3'-AC-5')
hs.Ri.MKI67.13.1	MKI67 knockdown	DsiR sense DsiR antisense	5'-GCAUUGAAGGAAUCUGCAAAACAGA-3' (3'-UU-5')
hs.Ri.MKI67.13.2	MKI67 knockdown	DsiR sense DsiR antisense	5'-GUCAGAAGGCAACACUACUACAACA-3' (3'-UC-5')
hs.Ri.GNL2.13.2	GNL2 knockdown	DsiR sense DsiR antisense	5'-CCCCUCACAUUGAAACUUACCUGAA-3' (3'-AA-5')
hs.Ri.GNL2.13.3	GNL2 knockdown	DsiR sense DsiR antisense	5'-ACAAAGGUCUGGCAGUAUAUUACTT-3' (3'-UU-5')
NC-1	Knockdown Negative control		Cat. #51-01-14-03

Each parenesis shows 2 bases overhang at 3' end of the antisense RNA. All were purchased from IDT.

Supplementary Table 3. **Quantitation of reciprocal IP yield.**

	Recovery by IP per IgG (relative)				
IB:	IP: nIgG	IP: ELMSAN1	IP: MDN1	IP: MKI67	IP: YTHDC2
ELMSAN1	0.07	1	0.36	**0.82**	0.64
MDN1	0.18	0.64	1	**1.16**	1.04
MKI67	0.02	**0.15**	**0.21**	**1**	**0.28**
YTHDC2	0.02	0.3	0.39	**0.85**	1
GNL2	< 0.01	**0.18**	**0.34**	**1**	**0.73**

The values can be compared across a row.
