# Supplementary material for: RNAmetasome network for macromolecule biogenesis in human cells
Source: Commun Biol. 2021 Dec 15;4:1399. doi: 10.1038/s42003-021-02928-y (PMC8674265; doi:10.1038/s42003-021-02928-y)
Supplement: Supplementary file 3 — Description of Additional Supplementary Files [file 42003_2021_2928_MOESM3_ESM.pdf]

Description of Additional Supplementary Files

File name: Supplementary Data 1-5
Description:

Supplementary Data 1: Inhibition of nuclear protein binding to the *IGFBPL1* promoter sequence by the CPN domain cluster. Y axis shows binding of nuclear proteins inhibited by the presence of the CPN domain, and X axis shows enrichment of proteins in the CPN domain-free nuclear extracts. Both axes present data as a ratio.

Supplementary Data 2: Enrichment of proteins by IP with the anti-ELMSAN1 antibody. Protein complexes by two independent IP (#1 and #2) were analysed by MS. “Fold” indicates the amount of protein enriched by the IP compared to the concentration in the nuclear extracts.

Supplementary Data 3: SAINT analysis of the IP-MS data (#1 and #2). The original MS data of the two experiments were analysed with SAINT.

Supplementary Data 4: SAINT analysis of another duplicate IP-MS data (#3 and #4). The original MS data were analysed with SAINT.

Supplementary Data 5: Comparison of RNAmetasome constituent proteins between HEK293T and Hela.cl1.
