# Supplementary material for: RNAmetasome network for macromolecule biogenesis in human cells
Source: Commun Biol. 2021 Dec 15;4:1399. doi: 10.1038/s42003-021-02928-y (PMC8674265; doi:10.1038/s42003-021-02928-y)
Supplement: Supplementary file 5 — Reporting Summary [file 42003_2021_2928_MOESM5_ESM.pdf]

nature research

Corresponding author(s): Shiro Iuchi

Last updated by author(s): November 23, 2021

Reporting Summary

Nature Research wishes to improve the reproducibility of the work that we publish. This form provides structure for consistency and transparency in reporting. For further information on Nature Research policies, see our Editorial Policies and the Editorial Policy Checklist.

Statistics

For all statistical analyses, confirm that the following items are present in the figure legend, table legend, main text, or Methods section.

n/a	Confirmed	
☐	☒	The exact sample size (*n*) for each experimental group/condition, given as a discrete number and unit of measurement
☐	☒	A statement on whether measurements were taken from distinct samples or whether the same sample was measured repeatedly
☐	☒	The statistical test(s) used AND whether they are one- or two-sided<br>*Only common tests should be described solely by name; describe more complex techniques in the Methods section.*
☒	☐	A description of all covariates tested
☒	☐	A description of any assumptions or corrections, such as tests of normality and adjustment for multiple comparisons
☒	☐	A full description of the statistical parameters including central tendency (e.g. means) or other basic estimates (e.g. regression coefficient) AND variation (e.g. standard deviation) or associated estimates of uncertainty (e.g. confidence intervals)
☐	☒	For null hypothesis testing, the test statistic (e.g. *F*, *t*, *r*) with confidence intervals, effect sizes, degrees of freedom and *P* value noted<br>*Give P values as exact values whenever suitable.*
☒	☐	For Bayesian analysis, information on the choice of priors and Markov chain Monte Carlo settings
☒	☐	For hierarchical and complex designs, identification of the appropriate level for tests and full reporting of outcomes
☒	☐	Estimates of effect sizes (e.g. Cohen's *d*, Pearson's *r*), indicating how they were calculated

Our web collection on statistics for biologists contains articles on many of the points above.

Software and code

Policy information about availability of computer code

Data collection	Mass spectra were acquired using a QQ Exactive HF-X mass spectrometer instrument from Thermo Fisher Scientific with the corresponding software (Tune 2.9 QF1) provided by the vendor.
Data analysis	Raw files were converted to mzXML using Raw File Reader (v3.0.77) provided by Thermo Fisher Scientific. Spectra were searched using SEQUEST 28. Search results were filtered using the LDA function in MASS Package in R as described in Huttlin et al. Cell 143, 1174-1189 (2010). All figures were made using Excel 2013 or R 3.4.2.

For manuscripts utilizing custom algorithms or software that are central to the research but not yet described in published literature, software must be made available to editors and reviewers. We strongly encourage code deposition in a community repository (e.g. GitHub). See the Nature Research guidelines for submitting code & software for further information.

Data

Policy information about availability of data

All manuscripts must include a data availability statement. This statement should provide the following information, where applicable:

- Accession codes, unique identifiers, or web links for publicly available datasets
- A list of figures that have associated raw data
- A description of any restrictions on data availability

The mass spectrometry proteomics data have been deposited to the ProteomeXchange Consortium via the PRIDE43 partner repository with the dataset identifier PXD024234. (43 after PRIDE is a superscript indicating a reference in our text.)

Field-specific reporting

Please select the one below that is the best fit for your research. If you are not sure, read the appropriate sections before making your selection.

☒ Life sciences ☐ Behavioural & social sciences ☐ Ecological, evolutionary & environmental sciences

For a reference copy of the document with all sections, see nature.com/documents/nr-reporting-summary-flat.pdf

Life sciences study design

All studies must disclose on these points even when the disclosure is negative.

Sample size	We empirically determine sample sizes by carrying out preliminary experiments and/or referring to other groups' sample sizes.
Data exclusions	We sometimes show our data on X-, Y- logarithmic scale diagram, where values below zero are automatically removed. Also, there are cases in which an extremely high value data could not be included in a diagram. If such a data had been included, the diagram would have been meaningless. However, what was excluded from the diagram can be traced from its table if readers wanted to know.
Replication	repeat typically twice.
Randomization	Randomization is not relevant to this work. Cell lines are used under defined experimental conditions.
Blinding	When I gave my samples to the collaborator, the samples were named only by alphabets.

Reporting for specific materials, systems and methods

We require information from authors about some types of materials, experimental systems and methods used in many studies. Here, indicate whether each material, system or method listed is relevant to your study. If you are not sure if a list item applies to your research, read the appropriate section before selecting a response.

Materials & experimental systems

n/a	Involved in the study	
☐	☒	Antibodies
☐	☒	Eukaryotic cell lines
☒	☐	Palaeontology and archaeology
☒	☐	Animals and other organisms
☒	☐	Human research participants
☒	☐	Clinical data
☒	☐	Dual use research of concern

Methods

n/a	Involved in the study	
☒	☐	ChIP-seq
☒	☐	Flow cytometry
☒	☐	MRI-based neuroimaging

Antibodies

Antibodies used	This information has been provided in Supplementary Table 1. Too many antibodies to describe here again.
Validation	I buy antibodies that suppliers provide photographs for specificity. When needed, specificity of antibodies has been verified by our experiments.

Eukaryotic cell lines

Policy information about cell lines

Cell line source(s)	HEK293T and Hela.cl1
Authentication	These are among the most often used cell lines in the field.
Mycoplasma contamination	No mycoplasma contamination.
Commonly misidentified lines (See ICLAC register)	*Name any commonly misidentified cell lines used in the study and provide a rationale for their use.*
